# Mechanical Properties and Degree of Conversion of a Novel 3D-Printing Model Resin

**DOI:** 10.3390/polym16243562

**Published:** 2024-12-20

**Authors:** Long Ling, Theresa Lai, Raj Malyala

**Affiliations:** Glidewell Dental, Irvine, CA 92612, USA; theresalai49@gmail.com (T.L.); raj.malyala@glidewelldental.com (R.M.)

**Keywords:** 3D printing, additive manufacturing, model resin, mechanical properties, degree of conversion, digital light processing (DLP)

## Abstract

The aim of this study was to evaluate the mechanical properties and degree of conversion of a novel 3D-printing model resin and compare it to eight commercially available model resins. An experimental resin formulated by our proprietary resin technology along with DentaModel, NextDent 2, KeyModel Ultra, Rodin Model, Die and Model 2, DMR III, LCD Grey, and Grey Resin were used in this study. Parallelepiped specimens (2 × 2 × 25 mm, n = 5) were printed and measured for their flexural strength (FS), flexural modulus (FM), and modulus of resilience (MR) in accordance with ISO-4049. Dumbbell-shaped specimens (Type V, n = 5) were printed to test tensile strength (TS) and elongation according to ASTM-D638. Barcol hardness (BH) was measured based on ASTM D2583 using broken tensile strength specimens. Izod-type test specimens (3.2 × 12.7 × 63.5 mm, n = 10) were printed, notched, and determined for impact strength according to ASTM D256-10. The degree of conversion was measured using FTIR (n = 5). Data were analyzed using one-way ANOVA and post hoc Tukey tests (*p* ≤ 0.05). The experimental resin exhibited a similar or significantly greater flexural strength (88.8 MPa), modulus of resilience (2.13 MPa), tensile strength (54.4 MPa), and hardness (82.9) than most model resins (FS 62.6–90.1 MPa, MR 1.37–2.0 MPa, TS 36.3–54.6 MPa, BH 66.1–83.7). The elongation (6.2%) and impact strength (14.2 J/m) of the experimental resin are statistically the same as those of most resins (3.0–7.5%, 13.8–16.4 J/m). However, the experimental resin has a significantly lower flexural modulus (1.97 GPa) than most resins (2.18–3.03 GPa). The experimental resin exhibited a significantly higher degree of conversion (66.58%) than most resins (1.11–62.34%) for 40 s of light curing; however, a similar or higher value (84.87%) than most resins (72.27–82.51%) was obtained for 3D-printed objects. The newly formulated 3D-printing model resin exhibited adequate mechanical properties and degree of conversion, which is comparable to the commercially available 3D-printing model resin materials. The new 3D-printing model resin can be used for modeling applications in restoration, orthodontics, implants, and other cases.

## 1. Introduction

Currently, 3D printing has been introduced in dentistry for a lot of applications, such as modeling, implant templates, surgical guides, night guard/occlusal splints, dentures, clear aligners, and temporary restorations [1,2,3,4,5,6,7,8] due to the rapid development of 3D printing in dentistry in recent years [9,10,11,12]. Among the 3D-printing techniques, stereolithography (SLA), digital light processing (DLP), and liquid crystal display (LCD) are three of the most popular and promising 3D-printing technologies capable of reaching a higher resolution and better mechanical properties for dental applications [13,14,15]. They all use a photosensitive resin to create the prints layer by layer based on light-polymerized technology. Dental models are widely used in dental laboratories and clinical practices. In contrast to traditional dental models like plasters or stones, 3D-printed models can be built with more complex geometries, high accuracy, and better structural durability [1,16,17]. 3D-printed dental model resins are the most widely marketed dental 3D printing materials in digital dentistry and have many applications in restoration, orthodontics, oral surgery and implants, teaching, patient consultation, and so on, for example, crown and bridge models, clear aligner models, implant models, and diagnostic models [17,18,19]. Sufficient mechanical properties are one of the most important prerequisites for such applications, as mechanical properties like flexural and tensile strength and hardness will affect the performance of model applications, especially for printability and model accuracy. To accurately simulate the anatomical structure (the tooth structure and the soft tissue like occlusal surface and space between the tooth and the gingiva), adequate mechanical properties are required to obtain good printability and excellent accuracy and to store the models for some time for clinical records [1,20]. However, to the best of the authors’ knowledge, no research about the evaluation of mechanical properties of 3D-printing resin materials for dental models has been reported until now.

This study aims to evaluate the mechanical properties and degree of conversion of a novel 3D-printing dental model resin and compare it with other commercially available 3D-printing model resins and build the correlations among various mechanical properties. The hypothesis is that the new 3D-printing model resin material has favorable mechanical properties and degree of conversion, which are higher or comparable to the most used commercially available 3D-printing model resin materials.

## 2. Materials and Methods

### 2.1. Materials

The 3D experimental model resin was formulated utilizing our proprietary resin technology and is composed of monomers (such as ethoxylated bisphenol A dimethacrylate and urethane dimethacrylate), a photoinitiator [bis(2,4,6-trimethybenzoyl)-phenylphosphine oxide], a UV stabilizer/blocker [2-hydroxy-4-methoxybenzophenone and 2,5-bis(5-tert-butyl-2-benzoxazolyl)thiophene], an inhibitor [2,6-di-(tert-butyl)-4-methylphenol], and pigments. The homogeneous model resin was prepared by stirring the resin monomers with the additives using an overhead stirrer (IKA RW20 digital) for at least 2 h. Eight commercially available model resin materials were selected for comparison. Further information about the resin materials used in this study, obtained from the manufacturers, is shown in Table 1.

### 2.2. Flexural Strength (FS), Flexural Modulus (FM), and Modulus of Resilience (MR)

The flexural strength, flexural modulus, and modulus of resilience were determined with the three-point bending method according to the literature [20]. Parallelepiped test specimens (thickness × width × length = 2 × 2 × 25 mm, n = 5) were printed using a DLP printing system (Asiga 3D printer, MAX UV 405 nm, same as below) from resin materials. After 3D printing, the specimens were washed with isopropyl alcohol for 5 min in Form Wash, post-cured for 60 min in Form Cure (Formlabs, Somerville, MA, USA) (same as below), polished, and tested at a crosshead speed of 0.75 mm/min on an Instron 5564 universal testing machine.

### 2.3. Tensile Strength (TS) and Elongation at Break (El)

Dumbbell-shaped specimens (Type V, n = 5) were made by 3D printing and tested after polishing under the crosshead speed of 1.0 mm/min on a Shimadzu (AGS-X-10 KN-table top model, Kyoto, Japan) universal testing machine in accordance with the literature [20]. Tensile strength (σ) was calculated using the following formula:

σ = W/A_0_

where W = breaking load (Newton), and A_0_ = original cross-sectional area (mm^2^).

Elongation at break (%El or ε) as a percentage was expressed as follows:

%El = (L − L_0_/L_0_) × 100

where L is the distance between gage marks at any time, and L_0_ is the original distance between gage marks.

### 2.4. Barcol Hardness (BH)

Broken tensile strength specimens were used for Barcol hardness (n = 5, five indentations per specimen). The Barcol hardness number was measured with the Impressor (GYZJ-935, Barber-Colman, Rockford, IL, USA) as outlined in ASTM D2583 [21]

### 2.5. Impact Strength (IS)

Impact strength was determined by test method A according to ASTM D256-10 [22]. Izod-type test specimens (n = 10) were 3D printed (thickness × width × length = 3.2 × 12.7 × 63.5 mm), polished, notched (width = 10.16 ± 0.05 under the notch) with the Impact Specimen Notcher (Model 899), and tested with a Pendulum Impact Tester (Model IT504) (Tinius Olsen, Horsham, PA, USA). Impact strength was calculated by dividing impact energy (J) by the thickness (m) of the specimen.

### 2.6. Degree of Conversion (DC)

Degree of conversion was measured using a Bruker ALPHA FTIR spectrometer with a Diamond Crystal ATR (Bruker, Billerica, MA, USA) according to the literature [23]. Disk specimens (n = 5) in the mold (10 mm in diameter × 1 mm in thickness) were placed directly on the diamond crystal plate and light cured for 40 s in situ with a light intensity of approximately 1100 mW/cm^2^ and a broadband spectrum of 385–515 nm (Bluephase Style, Ivoclar Vivadent AG, Schaan, Liechtenstein). For the final 3D-printed model object, the broken tensile strength specimens were used to collect cured 3D-printed resin after polishing (1 mm in thickness, n = 5). The DC was determined based on the peak heights of aliphatic double-bond absorption around 1638 cm^−1^ and of the aromatic double bond around 1607 cm^−1^ (as the internal standard) as follows:

DC (%) = [1 − (A_p_/A_p0_)/(A_m_/A_m0_)] × 100

where A_p_ is the peak height of the cured resin (polymer), and A_m_ is the peak height of the uncured resin (monomer) at 1638 cm^−1^; A_m0_ and A_p0_ are the peak height at 1607 cm^−1^ before and after the cure, respectively.

### 2.7. Statistical Analysis

Statistical analysis was performed using Minitab 21 statistical software (Minitab, LLC, State College, PA, USA). For various mechanical properties and degree of conversion, to evaluate the significance of the difference between groups (exp. resin and commercial resins as different variables), the data (mean and standard deviation) were analyzed with one-way ANOVA and Tukey’s post hoc test (pairwise comparison). The significance level was set at α = 0.05. Pearson’s correlation between different mechanical properties was analyzed by simple linear regression.

## 3. Results

**Figure 1:**
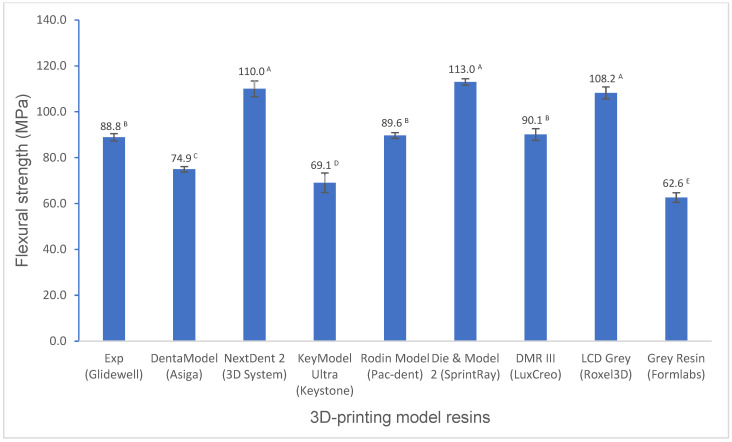
Flexural strength of 3D-printing model resin materials (values with the same superscript are not significantly different (*p* > 0.05).

**Figure 2:**
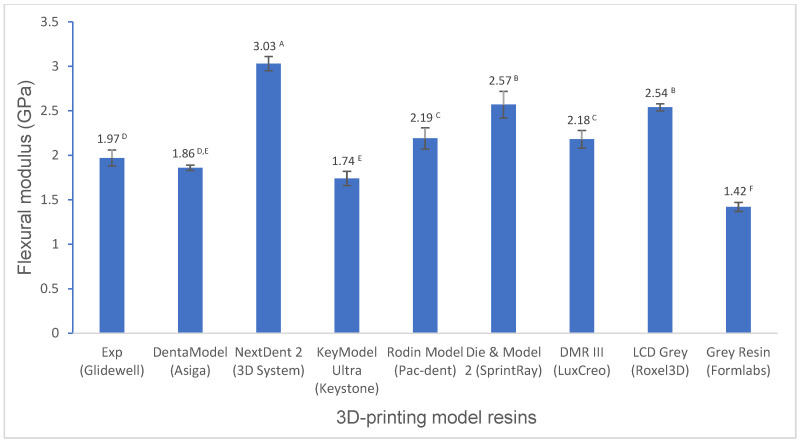
Flexural modulus of 3D-printing model resin materials (values with the same superscript are not significantly different (*p* > 0.05).

**Figure 3:**
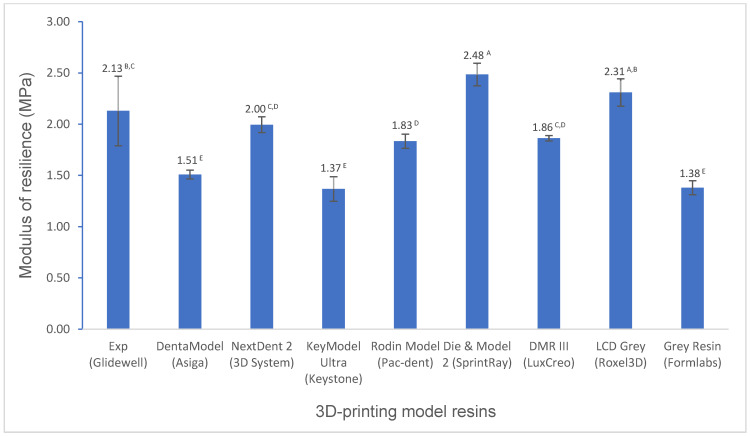
Modulus of resilience of 3D-printing model resin materials (values with the same superscript are not significantly different (*p* > 0.05).

**Figure 4:**
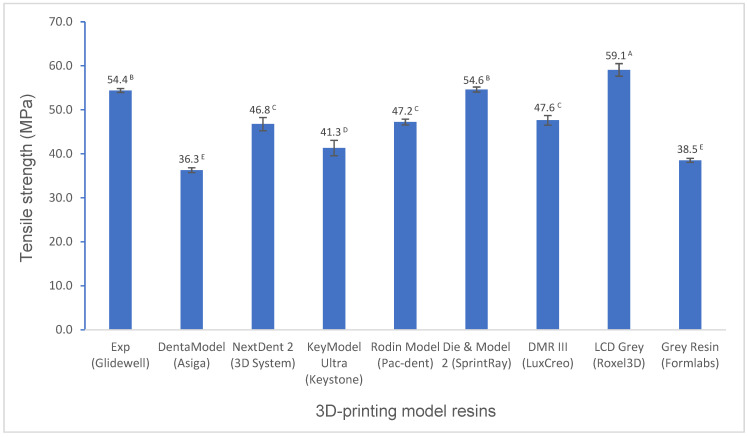
Tensile strength of 3D-printing model resin materials (values with the same superscript are not significantly different (*p* > 0.05).

**Figure 5:**
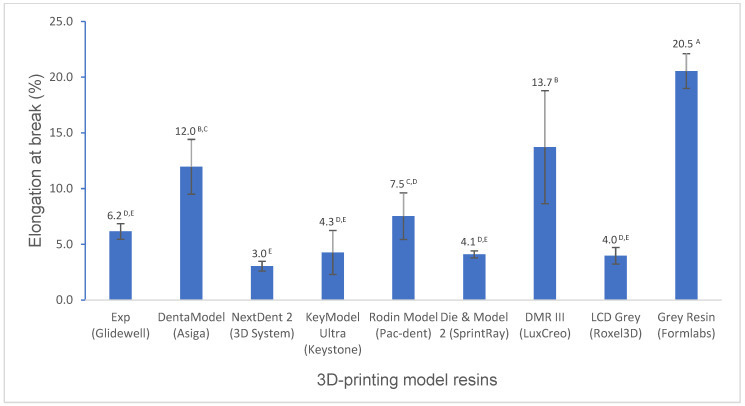
Elongation at break of 3D-printing model resin materials (values with the same superscript are not significantly different (*p* > 0.05).

**Figure 6:**
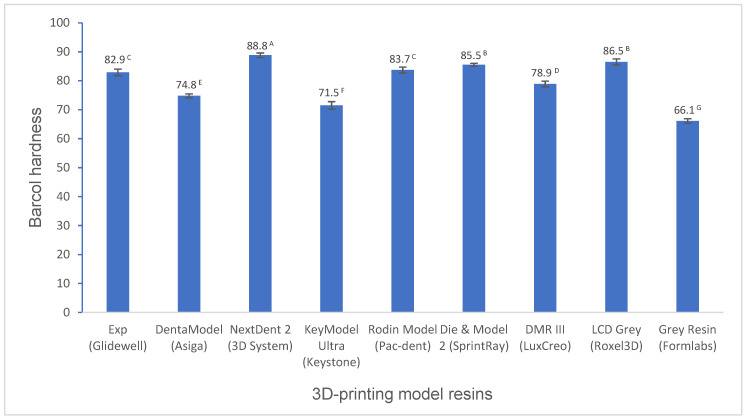
Barcol hardness of 3D-printing model resin materials (values with the same superscript are not significantly different (*p* > 0.05).

**Figure 7:**
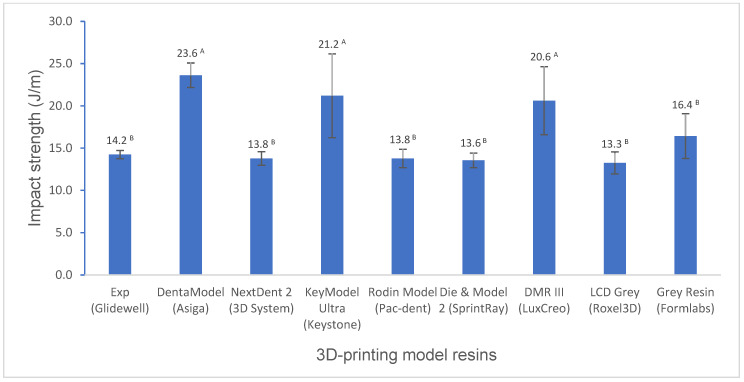
Impact strength of 3D-printing model resin materials (values with the same superscript are not significantly different (*p* > 0.05).

**Figure 8:**
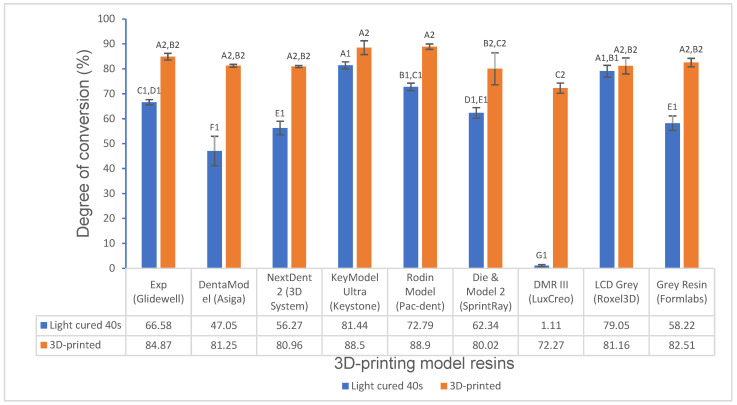
Degree of conversion of 3D-printing model resin materials (values with the same superscript are not significantly different (*p* > 0.05).

Various mechanical properties and degree of conversion at two curing conditions for experimental and commercially available model resin materials were shown in Figure 1, Figure 2, Figure 3, Figure 4, Figure 5, Figure 6, Figure 7 and Figure 8. The data were analyzed by one-way ANOVA and Tukey tests (*p* ≤ 0.05). Means with the same superscript are not statistically different between the tested groups in Figure 1, Figure 2, Figure 3, Figure 4, Figure 5, Figure 6, Figure 7 and Figure 8.

For flexural properties (flexural strength, flexural modulus, and modulus of resilience), the experimental resin showed a significantly higher (*p* < 0.001) or similar (*p* > 0.05) flexural strength than most model resins except NextDent 2, Die and Model 2, and LCD Grey (Figure 1), a significantly higher flexural modulus than DentaModel, KeyModel Ultra, and Grey Resin (*p* < 0.001), a lower flexural modulus than the other resins (*p* < 0.001) (Figure 2), and a significantly higher modulus of resilience than most resins except Die and Model 2 and LCD Grey (*p* < 0.001) (Figure 3).

The tensile strength of the experimental resin is statistically higher than that of most resins (*p* < 0.001), except it is lower than that of LCD Grey (*p* < 0.001) and similar to that of Die and Model 2 (*p* > 0.05) (Figure 4). However, the elongation at the break of the experimental resin is similar to those of most resins (Figure 5).

Barcol hardness was determined using the Impressor (a digital Barcol hardness tester, unit is 0–100) by the resistance to penetration by the metal tip from the surface of the materials. The experimental resin has statistically higher Barcol hardness than DentaModel, KeyModel Ultra, DMR III, and Grey Resin (*p* < 0.001), lower Barcol hardness than NextDent 2, Die and Model 2, and LCD Grey (*p* < 0.001), and similar Barcol hardness to Rodin Model (*p* > 0.05) (Figure 6).

The impact strength of the experimental resin is statistically the same as that of most resins and lower than that of DentaModel, KeyModel Ultra, and DMR III (Figure 7).

The experimental resin exhibited a significantly higher degree of conversion than DentaModel, Next Dent 2, Die and Model 2, DMR III, and Grey Resin (*p* < 0.001) and a lower degree of conversion than KeyModel Ultra, Rodin Model, and LCD Grey for 40 s light curing; however, for 3D-printed objects, the experimental resin showed similar results as DentaModel, Next Dent 2, LCD Grey, and Grey Resin (*p* > 0.05) and higher degree of conversion than Die and Model 2 and DMR III (Figure 8).

Pearson correlation coefficient (R) between any two mechanical properties for all model resins are shown in Table 2. Very strong/strong or moderate correlations were observed among these mechanical properties (Pearson’s correlation coefficient R > 0.8 is very strong, >0.6 or 0.7 is strong, and >0.4 or 0.5 is moderate [24,25]).

## 4. Discussion

Adequate mechanical properties are important for dental model resin materials to obtain good printability, accuracy, and clinical treatment success [1,20]. It is necessary to understand various mechanical properties of model resin materials to evaluate the new 3D-printing model resin. Flexural properties, tensile strength, impact strength, and hardness were evaluated in this study, which are the most studied mechanical properties for 3D-printed dental materials by manufacturers and academic research [26,27,28].

The flexural properties, including the flexural strength, the flexural modulus, and the modulus of resilience, are crucial mechanical properties. Flexural strength is the ability of a material to resist deformation under bending forces and refers to the maximum stress a material can withstand before breaking. The flexural modulus represents the stiffness of a material in resisting bending or flexing. The modulus of resilience represents a material’s ability to absorb and release energy without permanent deformation. Significant differences in flexural strength, flexural modulus, and modulus of resilience were observed among the tested model resins. The experimental resin had significantly higher flexural strength than DentaModel, KeyModel Ultra, and Grey Resin, was statistically the same as Rodin Model and DMR III, and had significantly lower flexural strength than NextDent 2, Die and Model 2, and LCD Grey (Figure 1). Flexural modulus had similar performance as flexural strength (Figure 2), NextDent 2, Die and Model 2, and LCD Grey exhibited higher flexural modulus than the others, which indicated that NextDent 2, Die, &Model 2, and LCD Grey are more rigid than the others. High rigid materials may be more brittle. For example, Die and Model 2 is more brittle than the experimental resin, as evidenced by our falling test, and this was often observed in Glidewell Laboratories (the largest dental laboratory with more than 5000 employees in the world) (easily broken on the floor when falling). The experimental resin exhibited a significantly higher flexural modulus than DentaModel, KeyModel Ultra, and Grey Resin (Figure 2). However, the experimental resin exhibited a significantly higher modulus of resilience than most resins except Die and Model 2 and LCD Grey (Figure 3). The modulus of resilience was determined by both the flexural strength and flexural modulus, which have opposite influences on the modulus of resilience. High flexural strength and low flexural modulus produce a higher modulus of resilience. For example, Die and Model 2 and LCD Grey have similar flexural strength as NextDent 2; however, NextDent 2 has a significantly higher flexural modulus than Die and Model 2 and LCD Grey, resulting in the modulus of resilience of NextDent 2 being significantly lower than that of Die and Model 2 and LCD Grey and even lower than that of the experimental resin.

Tensile properties are other important mechanical properties. Tensile strength is the maximum stress that a material can withstand when pulled from opposing sides before breaking. Elongation at break is a material’s resistance to breaking when stretched, which indicates how much a material can elastically and plastically deform before failure. LCD Grey and Die and Model 2 exhibit higher tensile strength, like their flexural strength. However, NextDent 2 did not show higher tensile strength like its flexural strength due to its high flexural modulus value (high stiffness) (Figure 4). The tensile strength of the experimental resin is statistically higher than those of most resins but is lower than that of LCD Grey and similar to that of Die and Model 2 (Figure 4). However, the elongation at break of the experimental resin is similar to that of most resins (Figure 5). Generally, high-strength materials that are brittle and hard typically feature a low elongation at break, like NextDent 2, which means that these materials exhibited little deformation when the stress is applied, while flexible and ductile materials have a high elongation and are less likely to break, like Grey Resin [27,28].

Surface hardness is the relative resistance of a material to an external indentation force. Surface hardness of the model resin materials was determined with Barcol hardness. Barcol hardness is a simple and rapid way to measure the surface hardness for an initial screen of mechanical strength. The model resin materials having higher flexural strength and modulus, like NextDent 2, Die and Model 2, and LCD Grey, showed significantly higher hardness than other resins. The model resin materials with a lower flexural strength and modulus, like DentaModel, KeyModel Ultra, and Grey Resin, showed lower hardness, which was supported by the high positive correlations between hardness and flexural strength (R = 0.9424) and hardness and flexural modulus (0.9145) (Table 2). DMR III has similar flexural strength and modulus results to the experimental resin; however, it showed lower hardness results than the experimental resin. Generally, hardness has a positive relationship with flexural modulus for resin composite materials [29]. Our results found this similar relationship between hardness and flexural modulus for (3D printing) resin materials (without fillers).

Impact strength, also called impact toughness, is a material’s ability to absorb shock and impact energy without breaking when the load is suddenly applied to it. Unlike most common mechanical strengths like flexural strength and tensile strength, which involve the gradual application of force until the material breaks, impact strength involves the near-instantaneous implementation of load which causes the material to absorb the energy. The Izod impact test was adopted to measure impact strength in this study. The experimental resin has significantly lower impact strength than DentaModel, KeyModel Ultra, DMR III, and Grey Resin and is statistically the same as the other resin materials. It seems that those resins, showing lower flexural strength and modulus or lower hardness like DentaModel, KeyModel Ultra, DMR III, and Grey Resin, have higher impact strength. The strong or moderate negative relationships between impact strength and flexural strength (R = −0.6485)/flexural modulus (R = −0.5377)/Bacol hardness (R = −0.6465) supported these results.

The abovementioned differences in mechanical properties among the tested model resin materials can be explained mainly by the differences in the composition and structure of the resin materials (resins and additives) [20,26,30,31], as they are printed and tested under the same condition. Generally, NextDent 2, Die and Model 2, and LCD Grey showed higher flexural properties (flexural strength, flexural modulus, and modulus of resilience), tensile strength and hardness, and lower elongation at break and impact strength; probably, they use some strong methacrylic oligomers or highly filled resin (Table 1). DentaModel contained a long-chain monomer (7,7,9(or 7,9,9)-trimethyl-4,13-dioxo3,14-dioxa-5,12-diazahexadecane-1,16-diyl bismethacrylate) and mono-methacrylate (Tetrahydrofurfuryl methacrylate). KeyModel Ultra and Grey Resin included urethane dimethacrylate or an oligomer and (meth)acrylate monomers, which may not be strong enough to support higher strength properties and showed some favorable flexible and toughness properties (Table 1). It should be noted that the manufacturers did not disclose the details of the resin composition, so these details are based on limited information and estimations. The experimental resin used the ethoxylated bisphenol A dimethacrylate (containing a rigid bisphenol A backbone which is not too viscous, unlike bisphenol A glycidyl methacrylate) and urethane dimethacrylate (containing some polar amide and ester bonds). These chemical structures provided strong strength support via some rigid backbones and some strong intermolecular interactions via polar bonds, resulting in relatively strong strength properties, for example, lower flexural strength and modulus than NextDent 2, Die and Model 2, and LCD Grey, and higher flexural strength and modulus than DentaModel, KeyModel Ultra, and Grey Resin.

The degree of conversion (DC) represents what percentage of monomers were converted into the polymers, indicating the curing efficiency of the resin monomers. The degree of conversion has a great influence on physical properties, mechanical properties, and biocompatibility [13,32]. FTIR is the most common technique that is widely used to measure the DC in most studies due to its sensitivity to specific functional groups involved in polymerization reactions [23]. The degree of conversion of all resin materials was investigated for 40 s light curing and 3D-printed objects, respectively. There were significant differences in the DC for 40 s light curing among these resin materials; for example, the experimental resin exhibited a significantly higher degree of conversion than DentaModel, Next Dent 2, Die and Model 2, DMR III, and Grey Resin and a lower degree of conversion than KeyModel Ultra, Rodin Model, and LCD Grey (Figure 8). However, there were no significant differences in the DC for 3D-printed objects among these resin materials except for DMR III. The DC for 40 s light curing can easily detect the DC differences among the resin materials and shows the high curing efficiency that the resin materials have, which mainly depends on the chemical structure and composition of the resins, like the monomers, initiator, inhibitor, and UV stabilizer/blocker [13,23]. This information can be useful for setting 3D-printing parameters and formula development. For example, a high-DC monomer (triethylene glycol dimethacrylate) and a highly active photoinitiator [bis(2,4,6-trimethybenzoyl)-phenylphosphine oxide] were used in our experimental resin [33,34], which made some contributions to the higher DC of the experimental resin. 3D-printed objects greatly enhanced the DC of the resin materials to a high level (about or over 80%), similar to the DC of other 3D-printed resins as reported by Perea-Lowery et al. [35], because curing condition (curing time, mode (layer by layer), layer thickness, energy, etc.) during 3D printing and post-curing play an important role in increasing the DC [35]. DMR III showed a very low DC for 40 s light curing (it is somewhat viscous which can be visually seen after 40 s light curing) probably due to its initiator system and inhibitor/UV stabilizer, etc.; however, its DC can jump to about 73% under the 3D-printing condition like post-curing. The DC of the resin materials can be increased to obtain favorable mechanical strength by optimizing the curing condition/profile. However, mechanical properties were affected by not only the DC but also other factors, as mentioned above, such as the internal structure and composition of the resins, the manufacturing process, etc. [20,26,30,32].

There are some descriptions about the correlations between the different mechanical properties for resin composite materials [29,36]. To the best of our knowledge, however, there is no or little report about such correlations for (3D-printing) resin materials. Based on nine 3D-printing resin materials, a Pearson’s analysis showed very strong or strong positive correlations between flexural strength and flexural modulus (R = 0.9424), flexural strength and the modulus of resilience (R = 0.9190), flexural strength and Bacol hardness (R = 0.9429), and flexural strength and tensile strength (R = 0.7925) but strong or moderate negative correlations between flexural strength and elongation (R = −0.6759) and flexural strength and impact strength (R = −0.6485), respectively. The relationships between flexural strength, flexural modulus, and Barcol hardness are similar to a study by Lee et al. for acrylic denture base resins (R = 0.75, 0.86) [37]. The relationships between different mechanical properties for (3D-printing) resin materials may be similar to or may be different from that of resin composite materials, as the mechanical properties of resin composites depends on both resins and fillers. For example, the moderate correlation between tensile strength and elongation at break (R = 0.5932) is in line with our recent report about light-body VPS impression materials (one kind of resin composites) (R = −0.5428) [38]; weak correlations were observed between flexural strength and the flexural modulus (R = 0.40), flexural strength and hardness (R = 0.39), and hardness and impact strength (R = 0.40) for contemporary resin composites [39]. The degree of such correlations between different mechanical properties depends on material categorization and composition [29,40].

This study evaluated various mechanical properties of a new 3D-printing model resin and eight other commercially available model resin materials to ensure they have adequate mechanical properties for digital dental model applications with good accuracy. However, the limitation is that this study did not include polymerization shrinkage and dimensional accuracy which also affects the accuracy of 3D-printed models. In addition, the same 3D-printing profile was used for different model resin materials to print the testing specimens, which may slightly affect mechanical properties. Future work will focus on shrinkage and dimensional accuracy, which is on-going and will be reported later.

## 5. Conclusions

The experimental model resin exhibited higher or similar values for mechanical properties and degree of conversion compared to most commercially available 3D-printing model resin materials. For example, the values of the flexural strength (88.8 MPa), modulus of resilience (2.13 MPa), tensile strength (54.4 MPa), and hardness (82.9) were similar or superior to those of the flexural strength (62.6–90.1 MPa), modulus of resilience (1.37–2.0 MPa), tensile strength (36.3–54.6 MPa), and hardness (66.1–83.7) of most model resins. A higher degree of conversion (66.58% for 40 s light curing and 84.87% for 3D-printed objects) for the experimental model resin made certain contributions to its adequate mechanical properties. Therefore, the hypothesis has been proven. Very strong/strong or moderate correlations (Pearson’s correlation coefficient R of 0.9429–0.5838) were observed between different mechanical properties, which provide a potential quick and simple way to estimate other mechanical properties based on some identified mechanical properties for (3D-printing) resin materials. The favorable mechanical properties of the novel 3D printing resin provide strong support for the obtainment of good printability and accuracy for 3D-printed dental models and make it suitable for modeling in restorative dentistry, orthodontic dentistry, implant dentistry, and so on.

## Figures and Tables

**Table 1:** 3D-printing model resins used in this study.

Material	Manufacturer	Color	Resin Composition
Exp. Model resin	Glidewell (Irvine, CA, USA)	Tan	Methacrylate monomers, urethane dimethacrylate, photoinitiator, UV stabilizer/blocker, BHT, pigments
DentaModel	Asiga (Alexandria, Australia)	Light beige	7,7,9(or 7,9,9)-trimethyl-4,13-dioxo3,14-dioxa-5,12- diazahexadecane-1,16-diyl bismethacrylate, Tetrahydrofurfuryl methacrylate, Diphenyl(2,4,6-trimethylbenzoyl) phosphine oxide
NextDent 2	3D System (Rock Hill, SC, USA)	Peach	Methacrylic oligomers, phosphine oxides, pigments
KeyModel Ultra	Keystone (Myerstown, PA, USA)	Ivory	Urethane oligomer, acrylate monomers, photoinitiator, titanium dioxide
Rodin Model	Pac-dent (Brea, CA, USA)	Pale yellow	Methacrylic esters, photoinitiators
Die and Model 2	SprintRay (Los Angeles, CA, USA)	Tan	Methacrylate monomers, methacrylate oligomers, photoinitiators, pigments
DMR III	LuxCreo (Belmont, CA, USA)	Orange	Not available
LCD Grey	Roxel3D (Orange, CA, USA)	Grey	Methacrylate monomer(s), photoinitiators, pigments
Grey resin	Formlabs (Somerville, MA, USA)	Grey	Urethane dimethacrylate, methacrylate monomer(s), photoinitiator(s), pigments

**Table 2:** Pearson’s correlation coefficient matrix of 3D-printed model resins.

	FS	FM	MR	BH	TS	El	IS
Flexural strength (FS)	1.00						
Flexural modulus (FM)	0.9424	1.00					
Modulus of resilience (RM)	0.9190	0.7396	1.00				
Barcol hardness (BH)	0.9429	0.9145	0.8577	1.00			
Tensile strength (TS)	0.7925	0.5942	0.9205	0.7688	1.00		
Elongation at break (El)	−0.6759	−0.6890	−0.5838	−0.7432	−0.5932	1.00	
Impact strength (IS)	−0.6485	−0.5377	−0.7022	−0.6465	−0.7233	0.4022	1.00

## Data Availability

Data are contained in this article.
